# Short- to long-term follow-up of total femoral replacement in non-oncologic patients

**DOI:** 10.1186/s12891-016-1355-6

**Published:** 2016-12-12

**Authors:** Andreas Toepfer, Norbert Harrasser, Isabel Petzschner, Florian Pohlig, Ulrich Lenze, Ludger Gerdesmeyer, Dominik Pförringer, Marcel Toepfer, Marc Beirer, Moritz Crönlein, Ruediger von Eisenhart-Rothe, Heinz Mühlhofer

**Affiliations:** 1 Department of Orthopedics and Sports Orthopedics, Technical University of Munich, 81547 Munich, Germany; 2 Sportmedizin Zürich, Schulthess Klinik, Zürich, Switzerland; 3 Department of Orthopaedic Surgery and Traumatology, University of Schleswig, Holstein, Germany; 4 Department of Trauma Surgery, Technical University of Munich, Munich, Germany; 5 Department of Nephrology and Dialysis, Klinikum Weilheim, Weilheim, Germany

**Keywords:** Total femoral replacement, Revision arthroplasty, Infection, Non-oncologic megaprosthesis

## Abstract

**Background:**

Compromised bone stock and heavily impaired structural integrity after multiple endoprosthetic revision surgeries can lead to a comparable condition as encountered in musculoskeletal tumor surgery. Total femoral replacement (TFR) can restore femoral integrity and allow patients to resume ambulation. Even though several authors reported their results of TFR, so far many questions are still on debate: Which patients are at risk to experience low functional outcome? Do complications and clinical outcome after TFR depend on the indication for the surgery (e.g. periprosthetic fractures or aseptic loosening) or the age of the patients? The purpose of the present study was to compare complication rates after TFR performed with modular total femur prosthesis MML (Fa. ESKA/Orthodynamics) in patients without malignant disease.

**Methods:**

We conducted a retrospective chart review and functional investigation of patients treated with a TFR for non-oncologic conditions from 1995 to 2015 and a minimum follow-up of 2 years. Complications were recorded according to the Henderson-Classification; outcome was evaluated with established clinical scores. The indication for TFR was periprosthetic fracture (Group A, *n* = 11) or aseptic loosening (Group B, *n* = 7) with massive bone defect of the femur deemed unsuitable for conventional arthroplastic or biologic reconstruction.

**Results:**

Eighteen patients matched the inclusion criteria and could be investigated clinically after a mean follow-up of 80 months (range: 28–132). Before TFA, all patients had previously undergone multiple operations (range: 1–8). The overall failure rate for any reason was 72% (*n* = 13/18), leading to a total of 37 surgical revisions with total exchange of TFR in 22% (*n* = 4/18). Most common failure mechanism was Type I (soft tissue), followed by Type IV (infection) and Type III (mechanical failure). According to Enneking’s functional evaluation method (MSTS-Score), the function ranged from 1 to 15 with a mean of 10 ± 4 out of 30.

**Conclusion:**

TFR is a salvage procedure to restore mechanical integrity and regain functional ability after extensive femoral bone loss. Outcome of the patients in the present study did mainly depend on the age at reconstruction and not on the indication for TFR.

## Background

**Fig. 1:**
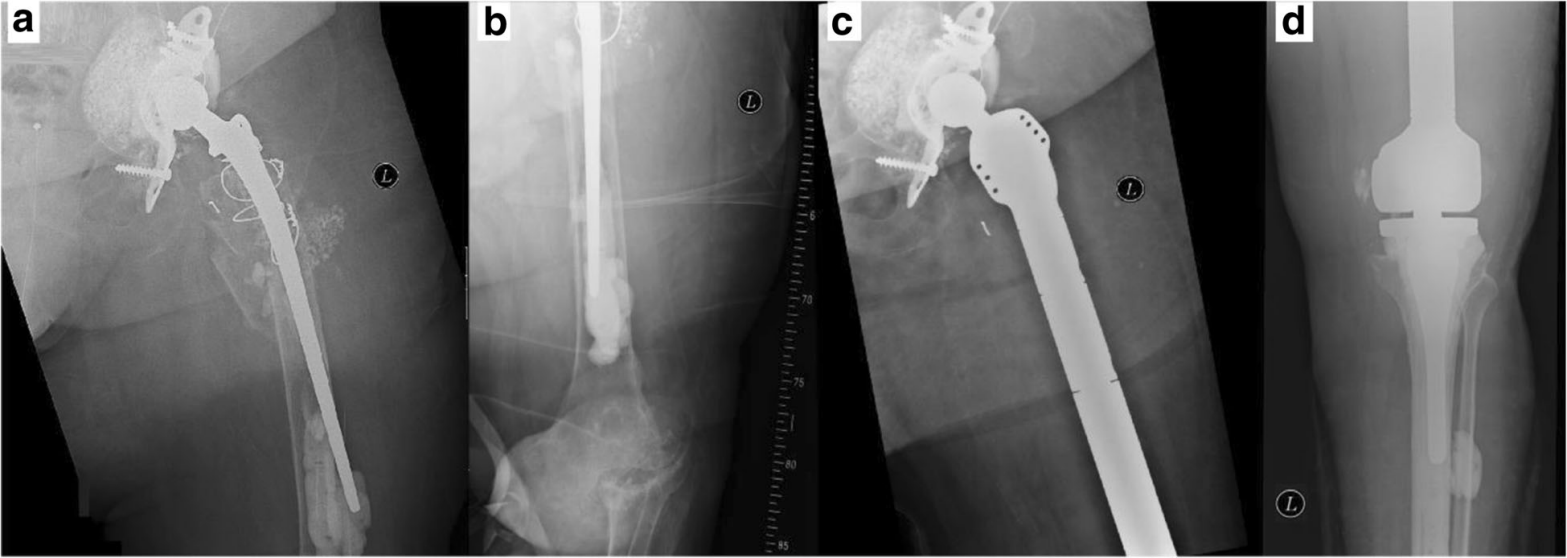
**a**, **b** X-ray of a patient with periprosthetic fracture at the level of the left femur and concomitant destructive osteoarthritis of the knee. **c**, **d** Reconstruction of femoral integrity was performed with total femoral replacement with MML-Prosthesis (acetabular cup was left in place due to no signs of loosening)

With increasing numbers of primary total hip and total knee arthroplasty, largely attributed to demographic changes in our society, endoprosthetic revision surgeries will become even more important in orthopedic surgery. Yet it is not only the sheer number but also the severity of revisions with massive, segmental bone loss and highly compromised soft tissues that will represent a major challenge to the future of joint replacement (Fig. 1).

In the lower extremity, the primary function is to support body weight and allow ambulation, durable reconstruction of bone defects after multiple revision surgeries is of major interest to preserve its function. Many surgical options for the reconstruction and stabilization of massive bone defects have been described and include, amongst others, the use of tumor endoprostheses [1]. The success of this procedure has come a long way, from custom-made monobloc devices to modular megaprostheses using the most modern implant techniques and surface technologies, including antibacterial coatings [2]. The most extreme use of modular megaprosthetics is considered to be the replacement of the whole femur, including the hip and knee joint. Total femoral replacement (TFR) can restore femoral integrity and allow patients to resume ambulation, although at a compromised level. Nevertheless, this compromised functional capacity is deemed superior to that achieved after hip exarticulation. There have been some reports analyzing functional outcome after treatment with TFR for aggressive musculoskeletal tumors [3–8], but only few for non-oncologic indications [1, 9–12].

The aim of this study was to retrospectively review the mid-term results after non-oncologic TFR for severe bone loss from one orthopedic center (Technical University Munich, Munich, Germany).

## Methods

This study was reviewed and approved by the Medical Ethical Committee at the Klinikum rechts der Isar (Technical University of Munich) and research was carried out in compliance with the Helsinki Declaration. Written informed consent was obtained from all patients included in the study.

**Fig. 2:**
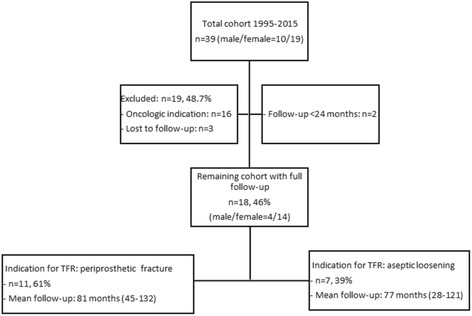
Constellation of total cohort and patients included in the study group

We retrospectively reviewed our institution’s database of patients who underwent a surgical procedure with resection of the femur due to bone tumors or failed revision arthroplasties and reconstruction using TFR from January 1995 to January 2015. Reconstruction was carried out with a modular total femur prosthesis (Type MML, Fa. ESKA/Orthodynamics, Luebeck, Germany) utilizing a standard ceramic head with 32 mm diameter, a non-cemented, spongy metal structured cup with conventional polyethylene liner (CL Type 2000 Plus or cranial socket, Fa. ESKA/Orthodynamics, Luebeck, Germany) and a hinged constrained total knee system (Fig. 1c, d). TFR was performed with the patient placed in a supine position and a lateral approach to the femur. After excision of the remaining femur and the prosthetic device (acetabular cup was left in place if no signs of loosening were evident), the total femur prosthesis was inserted with first preparation of the acetabulum, then the knee, and at the end connection of both parts with the diaphyseal modules. Hip abductor muscles were fixed to the trochanteric part of the TFR with non-resorbable suture material. Routinely, a 10-day antibiotic treatment with cefuroxime was given postoperatively. Antithrombotic prophylaxis with low-molecular-weight heparin was administered for six weeks. Physiotherapy was conducted for the first 3 months. It consisted of exercises aimed for increasing circulation to the legs and feet to prevent thrombosis. Muscle strengthening and mobilization of the hip was a focus of the therapy. Patients were allowed to perform exercises in the water after definite wound healing. According to the post-op standard, partial weight bearing within the first 4 weeks after surgery was allowed. We identified 39 patients (39 implants) with TFR. 16 patients were excluded due to an oncologic indication for their TFR. Of the 23 remaining patients, 3 were excluded due to unattainability, 2 due to follow-up of less than 24 months. Thus, 18 patients were included in our study for clinical assessment and survival analysis of the TFR (Fig. 2). Demographic data of the cohort are given in Table 1. Patients were subdivided into Group A and B according to the indication for TFR: Group A consisted of patients with a periprosthetic fracture (*n* = 11; mean age at reconstruction with TFR 77 years (±8 years), range 67–90), Group B had surgery for aseptic loosening (*n* = 7; mean age at reconstruction with TFR: 79 years (±7 years), range: 70–88). This subdivision was carried out on the assumption that indication for TFR might influence its outcome [13]. Patients were routinely followed after TFR at least once a year after the first year from reconstruction with TFR. Patients living far away from our clinic were contacted and asked to present for participating at the study. Hence, at the latest follow-up (Table 2), details of the postoperative course (i.e. complications), and scores analyzing the current function (MSTS score, Harris Hip Score (HHS), Oxford Knee Score (OKS), SF-12-health survey) were evaluated. VAS values are collected routinely prior every surgical intervention at our clinic, so that this score was available also prior reconstruction with TFR. All other functional scores were only collected at latest follow-up. Complications were classified according to the five modes of failure for megaprostheses proposed by Henderson et al. [14]: soft tissue failure (Type I), aseptic loosening (Type II), structural failure of implant and/or bone (Type III), infection (Type IV), and tumor progression (Type V).

**Table 1:** Demographics of patients after TFR

Demographic	Mean ± SD
Age (years)	78 ± 7 (range, 67–90)
Sex (Male/Female)	4/14
Height (cm)	162 ± 10
Weight (kg)	74 ± 11
BMI (kg/m^2^)	27.5 ± 4.6
Mean follow-up (months)	80 ± 28 (range, 28–132)
Side of TFR (Right/Left)	10/8

**Table 2:** Main data of patients regarding medical history and functional outcome according to MSTS-score. Indication for TFR was periprosthetic fracture (Group A) of aseptic loosening (Group B)

Group/Patient Number	Arthroplasty prior reconstruction with TFR	Age at reconstruction with TFR	Number of Revisions prior to TFR	Follow-up (months)	MSTS Score	
A/1	DFP and HP	76	3	132	15	Group A: 10 (±5)
A/2	DFP and HP	67	5	45	15	
A/3	PFP and KP	81	7	78	10	
A/4	DFP and HP	76	3	79	11	
A/5	Nail and HP	71	3	79	12	
A/6	HP	69	2	99	14	
A/7	HP and KP	70	7	87	13	
A/8	KP	84	1	54	4	
A/9	KP	86	8	45	1	
A/10	HP and KP	74	1	85	8	
A/11	Nail and KP	90	4	111	6	
B/12	HP and KP	79	1	58	8	Group B: 10 (±4)
B/13	KP	70	2	67	15	
B/14	DFP	85	2	121	5	
B/15	HP and KP	88	1	28	7	
B/16	HP	70	1	109	9	
B/17	HP	83	2	76	15	
B/18	HP and KP	77	3	80	10	
Mean		78	3	80	10	
Standard deviation		7	2	28	4	

*MSTS* musculoskeletal tumor society (MSTS-score is given as patient-specific mean score and group-specific mean score), *HP* hip prosthesis, *KP* knee prosthesis, *DFP* distal femoral prosthesis, *PFP* proximal femoral prosthesis

### Statistics

The survivorship analysis was performed using the Kaplan-Meier survivorship method. All data are reported as the mean and standard deviation, where applicable. Comparisons of patient-reported outcomes were performed using a *t*-test. Statistical significance was set at *p* < 0.05. Correlations between numerical data were done with linear regression analysis, and Pearson’s correlation coefficient (r) is reported. Statistical analysis was performed using SPSS 2.0 (IBM, Armonk, NY, USA).

## Results

### Indication for TFR

Prior to defect reconstruction with TFR all patients had a history of at least one orthopedic/traumatologic surgical procedure (i.e. nail, primary hip and/or knee prosthesis or mega-prosthesis). Specific indications for TFR of either group are given in Table 2. In Group A, 44 revision surgeries prior to TFR were reported in a total of 11 patients, leading to a total revision number of 4 ± 2 per patient (range: 1–8). In Group B, seven patients had a history of a total of 12 revision surgeries prior to their TFR, leading to a total revision number of 2 ± 1 per patient (range: 1–3).

### Complications

The average time from surgery to the development of a complication was 14 months (range: 1–105 months) with an average time to complication for Group A of 26 months (range: 11–60 months) and 6 months (range: 1–9 months) for Group B. Average time to occurrence of failures according to the Henderson-Classification varied depending on the different types. Type I was observed in 11 patients at an average time of 12 months (range: 4–54 months) after surgery; Type III was observed in 2 patients at an average of 34 months (range: 14–48 months) after surgery; Type IV was observed in 8 patients at an average of 50 months (range: 4–105 months) after surgery. A Type II failure was not observed in any of the patients. Type V failure (tumor recurrence) was not eligible for this cohort of non-oncologic patients, respectively. Overall, there were 28 implant-related complications according to Henderson in 13 patients with all of these being either Type I, III or IV failures (Table 3). Complications led to 37 revision surgeries (implant-related and implant-independent).

**Table 3:** Number of failures in this series as classified according to Henderson et al. [14]; Note: 28 complications were found in 13 patients (some patients had multiple failures)

Type of failure	Group A (*n* = 11)	Group B (*n* = 7)	Total number of complications
I (soft tissue failure)	3 dislocations 5 wound healing problems 1 arthrofibrosis	2 dislocations 6 wound healing problems 1 arthrofibrosis	5 11 2
II (aseptic loosening)	-	-	-
III (structural)	1 breakage of bolt	1 breakage of bolt	2
IV (infection)	3	5	8
V (tumor progression)	-	-	-
**Total**	**13**	**15**	**28**

### Analysis of complication-types

Type I Recurrent hip dislocations were observed in 5 patients (Group A: 3; Group B: 2), whereof all patients initially underwent closed reduction and conservative treatment. 2 patients had to undergo three revision surgeries subsequently (1× replacement of the head, 1× replacement of inlay and head, 1× cup replacement). Wound healing problems were observed in 11 cases (Group A: 5; Group B: 6) with the need of 11 surgical interventions in 10 patients. Conservative wound treatment was successfully performed in 1 case. Painful knee arthrofibrosis with a limited range of motion was observed in 1 case of either group: 1 patient (Group A) was revised with a knee arthrodesis, 1 patient (Group B) received successful joint mobilization (brisement forcé) under general anesthesia.
Type III In either group 1 patient sustained a mechanical failure of its TFR. In both patients, a failure of the hinge-mechanism of the fully constrained knee module with breakage of the bolt was observed. Both knees were revised with an exchange of the inlay and the hinge-mechanism.
Type IV Septic complications of the TFR were observed in 8 patients (Group A: 3; Group B: 5) with a total of 19 revision surgeries. One patient had to undergo only 1 revision, all others at least 3. Four patients needed total (all components), 4 patients partial exchange of the TFR. All patients had antibiotic treatment for 6–12 weeks after the last surgical intervention for infect eradication, no patient needed lifelong suppression therapy. The microbes detected were 6× *Staphylococcus epidermidis*, 1× *Propionibacterium acnes*, 2× *Staphylococcus aureus*, 2× *Enterococcus faecalis*, 1× *Micrococcus luteus* (some patients hat multiple bacteria).

### Implant survival analysis

Mean follow-up of patients was 80 ± 28 months (range: 28–132). Implant failure was defined as partial or complete exchange of the megaprosthesis due to implant-related complications. Implant failure was detected in 44% (*n* = 8/18) of cases at five years, and 56% (*n* = 10/18) al latest follow-up. Only 5 patients had an uneventful implant-survival without any kind of revision surgery throughout the study period.

### Clinical outcome

Overall, patients in this series had a mean VAS-score preoperatively of 5.9 (Group A: 5.9; Group B: 5.9) and postoperatively after TFR of 3.6 (Group A: 3.5, Group B: 3.6). The differences were significant (*p* < 0.001) comparing pre- and postoperative VAS-scores.

Clinical outcome data computed by MSTS score, HHS and OKS, as well as results of SF-12 analysis are given in Tables 2 and 4. There were no differences neither between the groups nor between the subgroups. Linear regression analysis between age/revisions prior TFR and clinical outcome (MSTS-score) revealed a moderate negative correlation for age (*r* = −0.69) and no correlation for prior revisions (*r* = −0.05).

**Table 4:** Functional outcome results of both groups

	Items	Group A [Value, (range)]	Group B: [Value, (range)]
HHS	Mean score	43 (16–70)	38 (21–60)
	*Pain*	23 (10–37)	20 (11–32)
	*Function*	9 (0–14)	6 (0–11)
	*Activity*	5 (0–10)	5 (1–10)
	*Contractures*	4 (3–4)	3 (3–4)
	*motion*	3 (2–4)	2 (2–4)
OKS	Mean score	14 (4–26)	17 (10–25)
SF-12	Physical subdomain	27 (21–36)	27 (22–35)
	Mental subdomain	38 (27–61)	38 (28–63)

*HHS* (harris hip score): <70: poor; 70–79: fair; 80–89: good; 90–100: excellent, *OKS* (oxford knee score): <19: poor; 20–29: fair; 30–39: good; 40–48: very good, *SF*-*12* (short form 12 health survey): healthy controls > 50

*p* < 0.05 = significant (*)

## Discussion

Reconstruction of massive bone defects of the femur after failed revision arthroplasty represents a challenge for orthopedic surgeons. TFR can restore femoral integrity and allow patients to resume ambulation, albeit at a compromised level. Usually, this reduced functional capacity is superior to that achieved after hip exarticulation [3]. This study analyzed the clinical and functional results of 18 TFRs of patients with a history of failed revision arthroplasty either due to periprosthetic fractures (Group A, *n* = 11) or aseptic loosening (Group B, *n* = 7). To our knowledge, there are no published case series using a standardized failure-mode classification with the presented numbers regarding TFRs performed with the MML-System (Fa. ESKA/Orthodynamics); therefore, we investigated the TFRs of a single centre University Institute for implant survival, functional outcomes and different types of complications.

A main finding of the present study is that no differences were observed in clinical outcome between the groups (Table 2). Subdivision of our cohort into two groups was performed on the assumption that indication might influence pre- and postoperative function [13]. Periprosthetic fractures cause acute onset of symptoms with the necessity of immediate treatment within a short period, whereas aseptic loosening usually goes along with a chronically painful dysfunction of the implant and the need for revision only in cases without improvement of symptoms after failed conservative treatment. In fact, we found that preoperative VAS-scores were similar in both groups without significant differences. Additionally, postoperative MSTS-scores, OKS and SF-12 health survey showed no differences between the groups. Functional outcome measurement according to the MSTS-Score revealed an average value of 10 (33%) in the present study and therefore the results were inferior in comparison to other studies investigating TFR after failed revision arthroplasty with average results between 59-77% (Table 5). Nevertheless, some specifications regarding individual cases of both groups are necessary for adequate interpretation of these values: Two patients (A8 and A9, Table 2) suffered from a strongly deteriorated health preoperatively with advanced dementia. Additionally, patients of the present study were significantly older compared to patients of other studies (Table 5). These factors influence the MSTS-score and might at least partially explain the differences between the groups. In fact, other authors found a significant influence of age at time of implantation and number of revisions prior to TFR on postoperative TFR outcome [13, 14]. In this regard, the results of the present study show some discrepancy with these findings. In our cohort, both groups were very similar regarding age (Group A: 77; Group B: 79; *p* > 0.05) but not regarding revisions prior to TFR (Group A: 4 ± 2; Group B: 2 ± 1; *p* < 0.05). In sum, indication (periprosthetic fracture vs. aseptic loosening) did not influence clinical outcome of TFR in the present study. Linear regression analysis between age/revisions prior TFR and clinical outcome revealed a moderate negative correlation for age, which means there is a tendency for older patients to go with low clinical outcome.

**Table 5:** Comparison of current study results with those of other studies involving femoral reconstruction with TFR for failed arthroplasty

Author	Number of patients	Follow-up [months]	Average age [years]	Functional outcome: postoperative (preoperative)	Revision-rate [%]	Survivorship of TFR	Complications requiring surgery (no. patients)
Amanatullah [9]	20	73	65	HHS: 65 (30)	30%	70% at 5 years	Infection (7), hip dislocation (5), limb length discrepancy (2), knee flexion contracture (1)
Berend [10]	58/59	58	74	HHS:71 (40)	30.5%	65% at 5 years	Infection (8), hip dislocation (7), tibial component loosening (2), acetabular component loosening (1)
Fontain [11]	12/14	90	63	MSTS: 59% (23%)	35.7%	NA	Hip dislocation (5), infection (3)
Friesecke [12]	81/100	59	68	MSTS: 77%	21%	NA	Infection (12), hip dislocation (6), prosthesis failure (3), patellar issues (2), hematoma (2), peroneal nerve palsy (1), delayed wound healing (1)
Lombardi [1]	50/75	42	73	HHS: improved by 14 points	30.7%	NA	Infection (11), hip dislocation (7), tibial component loosening (2), Acetabular component loosening (1), hematoma (1), periprosthetic fracture (1)
Current study	18/20	80	78	MSTS: 33% HHS: 41	72%	56% at 5 years	Infection (8), hip dislocation (2), wound healing problems (10), prosthesis failure (2), arthrofibrosis (2)

*Note*: Number of patients (x/y): number of patients included in study/number of total patients including drop-outs

*HHS* Harris hip score, *MSTS* musculoskeletal-tumor-society-score

HHS as a well-established hip score has so far only been used by *Berend* et al. to evaluate hip function in TFR [10]. In his series, an average value of 70 was found. In the present study, an overall score of 41 was detected with again no significant differences between the groups (Group A: 43; Group B: 38). Evaluation of TFR cannot be compared to results from primary or conventional revision THA. The OKS has so far not been evaluated for TFR. In the present study, a rather low average value (32%) was found, with again no significant differences between the groups (A: 30%; B: 35%). As with HHS, OKS seems not to be an appropriate tool to evaluate functional outcome after TFR. In summary, pain measures revealed a significant reduction of pain after TFR compared to preoperative values. Function with mobility is nevertheless reduced in both groups. This finding is supported by the physical SF-12 survey score (Table 4). The mental SF-12 survey scores were similar in both groups, stating good acceptance of the TFR. Patients of the present study communicated clearly that regain of partial mobility and reduction of pain are the most important items to achieve satisfaction after TFR. Other authors have already published this finding [10, 11].

**Fig. 3:**
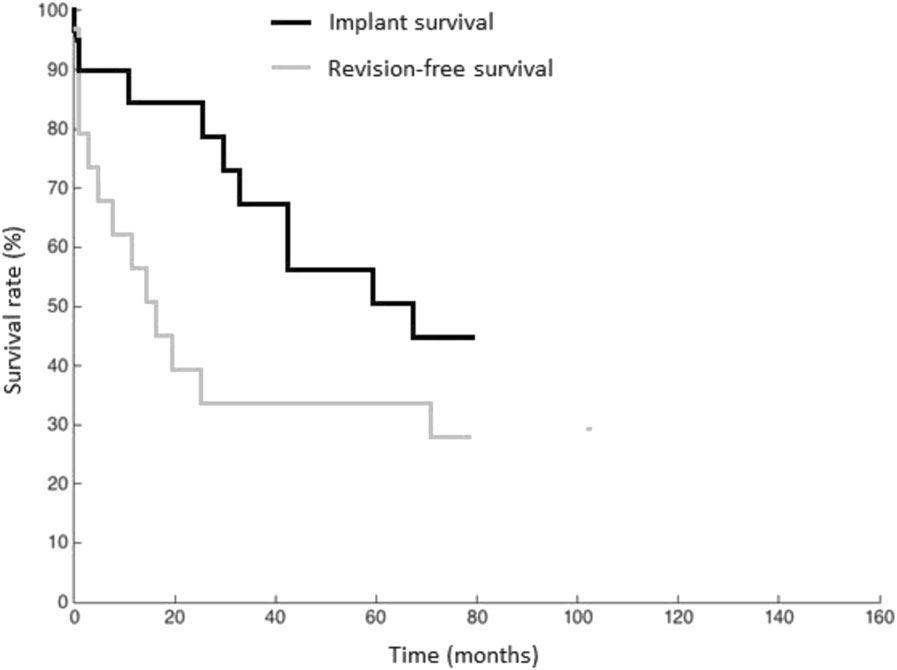
Kaplan-Meier survival analysis with TFR 5-year-implant-survival without exchange of any parts of the prosthesis of 56%, and TFR revision-free survival of 28%

Another main finding of the present study was a 72% overall complication rate in our patients treated with TFR (Fig. 3). It has to be stated that none of the included patients (*n* = 18) in the present study had a primary defect reconstruction with TFR. In fact, 56 revision surgeries were performed in the study population prior to implantation of TFR. This is important as postoperative complications after TFR might be at least partly related to prior surgical procedures. Despite the high complication rates, only 4 TFRs had to be completely replaced. Most of the postoperative revision surgeries had to be performed due to soft tissue problems (Type I failure) which were detected in 11 cases, necessitating 16 surgical revisions. The most common cause for Type I failure was aseptic wound dehiscence (*n* = 11), followed by hip dislocation (*n* = 5) and knee arthrofibrosis (*n* = 2). In the literature, reported rates vary between 0% and 45% [3–5, 7, 11] for Type I complications. It is known from large series with primary hip arthroplasty that 75% of dislocations occur within the first two months after implantation [15]. In our study, this was observed in 67% of dislocations. To prevent hip dislocation in cases of residual trochanteric bone or viable tendinous abductor structures we preferred direct attachment to the endoprosthetic implant with non-resorbable sutures. Promising results regarding hip stability can be obtained if tripolar cups are used [2, 3, 8]. Where applicable, tripolar cups are now performed routinely in our clinic to reduce rates of hip dislocation.

Aseptic loosening (Type II failure) in megaprostheses was reported in the literature with a rate ranging from 2.4 to 15.4% for cemented stems [16–19] and from 0% to 8% for cementless implants [20–23]. In our series, no aseptic loosening was found and therefore confirmed the data of the literature with a low incidence of this type of failure in TFR [5, 11]. Contrary to proximal or distal femoral prostheses (PFP/DFP), TFR implantation does not have to rely on diaphyseal stem fixation but uses common techniques of THA and (fully constrained) TKA with a standard acetabular cup and tibial meta-diaphyseal stem fixation. This might explain better results of TFR regarding aseptic loosening compared to PFR and DFR.

Structural failure (Type III failure) was observed in two patients in our series. The structural failure was a prosthetic breakage at the level of the hinged-knee joint. In both cases, the affected prosthetic parts were surgically exchanged and no further material failure was observed. In the literature, the incidence of prosthetic component breakage in megaprostheses ranges between 0% and 7.7%, with lower incidences in TFR compared to distal or proximal femoral replacement. Again, this might be accounted for by an absence of diaphyseal stems in TFR which are a known weak spot in modular megaprostheses [24]. Other authors conclude, reduced mobility and activity in this population as a reason for lower rates of structural failure probably due to lesser activity in this population [16, 18, 19, 21–23].

Type IV failure (deep infection) was observed in eight out of 18 patients (44%) in our series. These data are considerably higher compared to infection rates described in the recent literature with a reported range from 0 to 35% in TFR [3–5, 7–9, 11]. A possible explanation for these high infection rates is the number of revision surgeries prior to implantation of TFR (56 in 18 patients). Additionally, it has to be mentioned that all patients with deep infection of the TFR had a septic complication of their prosthesis prior to implantation of their TFR. It is known, that reinfection rate after cured deep infection is considerably higher than infection rates of primary prostheses [25]. Another explanation may be found considering the demographic data of our cohort showing that our patients are significantly older compared to studies with TFRs from oncologic patients [13]. Nevertheless, despite the high infection rates permanent eradication of infection was achieved in all cases. This might be due to our aggressive and early surgical interventions whenever deep infection was present leading to a total of 19 revisions in our cohort. In four patients complete exchange of TFR, in all other patients partial exchange of the modular parts of the TFR was performed. One patient had to undergo only one revision, all other patients needed multi-stage revision surgeries to eradicate infection. All patients had antibiotic treatment for 6–12 weeks after the last surgical intervention for infect eradication, no patient needed lifelong suppression therapy.

The implant-independent failure mechanism Type V (tumor progression/recurrence), was never observed in our cohort of patients, as defined in our inclusion criteria. For oncologic indications of TFR, authors describe rates between 5 and 20% [3–5, 7].

This study has some limitations that bear discussion. At first, the retrospective study design is subject to recall and selection bias. The number of patients is small and statistical analysis therefore difficult. Nevertheless, due to the rare indication for this procedure our series is comparable to studies published earlier. Secondly, this study lacks a control group. Thus, we cannot directly compare our results with other types of implants or biologic reconstructions.

## Conclusion

Preservation of lower limb integrity and immediate stability to allow early mobilization are the primary benefits of TFR. Nevertheless, patients requiring this salvage technique are usually severely debilitated and their associated perioperative morbidity must be taken into consideration. The retrospective analysis of our series confirmed the high incidence of implant-related complications and failures in TFR with infection and soft tissue failure as the most frequent modes of complications. Implant survival of 56% at 5 years was observed, and seems not to be affected by initial diagnosis for TFR (periprosthetic fracture vs. aseptic loosening). Non-oncologic patients who receive TFR as a form of end-stage revision arthroplasty have to be informed about the less favorable results compared to “conventional” revision joint arthroplasty and TFR for oncologic indications. The ultimate failure of TFR most commonly results from persistent periprosthetic infection and might eventually require hip exarticulation. Therefore, TFR requires very strict indications and potential benefits and burdens of this procedure have to be evaluated individually. Nevertheless, TFR sometimes remains the only viable option to allow functional limb salvage in rare cases of non-reconstructable periprosthetic fracture situations and extensive bone loss caused by repeated endoprosthetic revision surgeries.

## Abbreviations

HHS: Harris-Hip-Score
MSTS-Score: Musculoskeletal-Tumor-Society-Score
OKS: Oxford-Knee-Score
TFR/DFR/PFR: Total/distal/proximal femoral replacement
